# Strong Enhancement of Two-Photon Absorption and Emergence of Unusual Extinction Saturation in Silver Sulfide Quantum Dots Integrated with Gold and Silica Nanostructures

**DOI:** 10.1021/acsami.5c00984

**Published:** 2025-05-03

**Authors:** Marta Gordel-Wójcik, Radosław Kołkowski, Marcin Nyk, Marek Samoć

**Affiliations:** † Faculty of Chemistry, University of Wrocław, 14.p F. Joliot-Curie Street, 50-383 Wrocław, Poland; ‡ Department of Applied Physics, Aalto University, P.O.Box 13500, FI-00076 Aalto, Finland; § Institute of Advanced Materials, Faculty of Chemistry, Wrocław University of Science and Technology, Wyb. Wyspiańskiego 27, PL-50370 Wrocław, Poland

**Keywords:** Z-scan, nanomaterials, quantum dots, silver sulfide, Ag_2_S, nanoshells, plasmon resonance, gold nanoparticles, two-photon absorption, saturable absorption

## Abstract

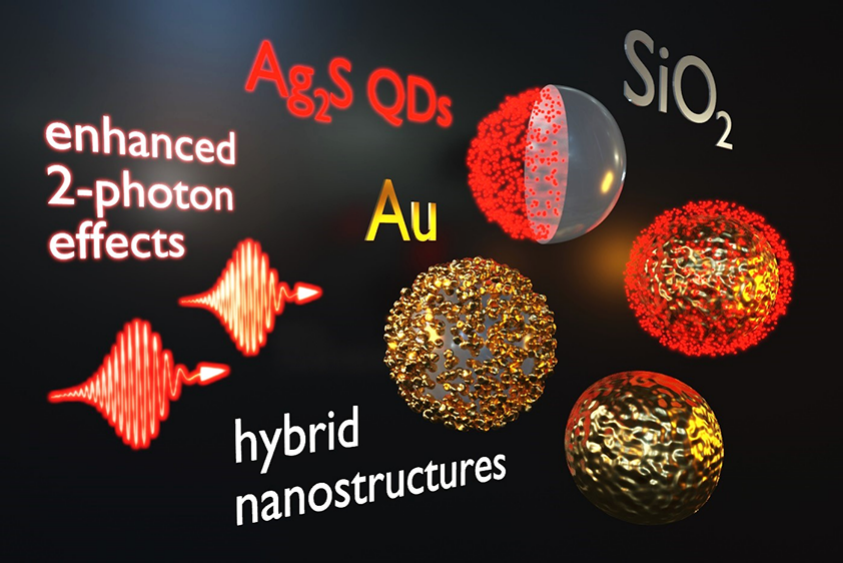

Hybrid nanosystems, such as those combining plasmonic, dielectric, and quantum-confined nanostructures, have long been of interest for enhancing and tailoring diverse light–matter interactions. Here, we present a series of hybrid nanomaterials exhibiting strongly enhanced nonlinear optical (NLO) properties, fabricated by combining silver sulfide quantum dots (Ag_2_S QDs) with silica and gold nanostructures. We studied their NLO properties (two-photon absorption and saturable absorption) in colloidal solutions over a wide spectral range (500–1600 nm) using the femtosecond Z-scan technique. Embedding Ag_2_S QDs into silica nanospheres gives rise to remarkable enhancement of two-photon absorption (up to a factor of 16 increase in the merit factor σ_2_/*M* compared to bare QDs), whereas covering such QD-doped silica nanospheres with gold nanoparticles or attaching the QDs to the surface of gold nanoshells (NSs) leads to even further enhancement (up to 73-fold increase in σ_2_/*M*), accompanied by a competing effect of saturable absorption. Furthermore, in the case of QD-doped silica spheres covered with a continuous gold layer, we observe a previously unreported saturation of extinction in the near-infrared region that follows an unusual intensity dependence, suggesting the involvement of two-photon absorption as the pumping mechanism. In addition to the experimental studies, we have performed numerical simulations, revealing the plasmonic origin of the observed spectral dependences of the NLO properties, with the underlying enhancement mechanisms involving local field enhancement and, possibly, also coupling between plasmon modes and QD excitons, giving rise to a double peak in the σ_2_ spectrum. Our findings demonstrate the unique potential of hybrid NLO nanomaterials combining quantum-confined, plasmonic, and dielectric components.

## 1 Introduction

Quantum dots (QDs) are broadly employed as alternatives for organic or organometallic dyes due to their numerous advantages, encompassing stability under light exposure, relatively narrow emission bandwidth, as well as broad absorption bands facilitating excitation across a wide spectral range.^1−4^ Another advantage lies in their nonlinear optical (NLO) properties, notably multiphoton absorption (especially two-photon absorption, 2PA) and saturation of one-photon absorption (1PA SAT).^5,6^ Such characteristics are highly desired for the development of novel functional materials, with wide-ranging applications in NLO devices such as passive Q-switches,^7,8^ up-conversion lasers,^9,10^ optical limiters,^11^ memories and neuromorphic computing systems,^12^ and infrared detectors.^13^

2PA materials also find applications in biological and medical imaging, particularly in enhanced imaging and process monitoring.^14−16^ Significant improvements can be achieved in spatial resolution and imaging depth^15,17^ in two-photon microscopy^18^ compared to traditional 1PA-based fluorescence microscopy, which can be crucial for imaging fine cellular structures and dynamics.^19^ Additionally, the use of 2PA leads to reduced photobleaching which enables longer observation times and more stable imaging.^20−22^

Of special interest are low-toxicity fluorescent markers absorbing and emitting light within the near-infrared (NIR) biological windows, the use of longer wavelengths (λ) improving the tissue penetration due to reduced absorption and scattering and mitigating autofluorescence.^23,24^ Such markers could employ QDs with efficient 2PA and high quantum yield of emission in the NIR range. We have demonstrated that silver sulfide (Ag_2_S) QDs show promising 2PA properties in the NIR range, while also exhibiting fluorescence emission within the low tissue absorption spectral range.^5^ However, their 2PA cross section (σ_2_) was found to be rather modest, 772 GM (Goeppert–Mayer units) at 875 nm. In order to compare those 2PA properties with other 2PA materials one needs to account for differences in QD sizes and weights. This is usually done by using molecular (or particle) weight normalized values, σ_2_/*M*, which for Ag_2_S QDs (M on the order of 4 × 10^4^–7 × 10^4^ g/mol), is found to be about 0.013 GM·mol·g^–1^. An order of magnitude higher values have been reported for cadmium-containing (more toxic) QDs,^25−27^ and even 2 orders of magnitude higher ones for some organics^28,29^ and organometallics.^30,31^

It has been found in many studies that Ag_2_S QDs exhibit excellent NIR photoluminescence, low toxicity, high photostability, and a long fluorescence lifetime, making them highly attractive for various photonic and biomedical applications.^32−35^ Their tunable energy band gap and solution processability further enhance their versatility in advanced technologies.^36^ However, as mentioned above, their moderate 2PA properties have limited their effectiveness in NLO applications. Therefore, developing an efficient strategy to enhance NLO processes in Ag_2_S QDs would be of significant interest.^5,37−39^ The literature suggests that the σ_2_ value can be increased by doping the QDs with metal nanoparticles (NPs)^40^ or metal ions,^41^ or by modifying their shapes.^26^ Another approach may involve fabrication of nanostructures containing more than a single QD: hybrid moieties exhibiting a cumulative absorption cross section, which may also be additionally enhanced through the interactions between different components of the nanostructure. This is possible, e.g., by using nanocontainers filled with QDs (see ref (42).) or by decorating the surface of a bigger nanoparticle (NP) with small QDs.^43^

Metal-shelled QDs exhibit unique optical and electronic properties due to the interaction between the semiconductor core and the surrounding metal shell. The presence of a metal shell, such as gold or silver, enhances charge carrier dynamics, modifies excitonic behavior, and introduces plasmonic effects that can significantly influence absorption and emission properties of QDs. These structures often demonstrate improved photostability, tunable band alignment, and enhanced NLO responses. Additionally, the metal shell can facilitate energy transfer processes and impact the density of electronic states, making metal-shelled QDs a subject of growing interest in fundamental nanomaterials research.^44−47^ In this context, there is considerable interest in developing hybrid nanomaterials that integrate optically resonant (e.g., plasmonic) nanostructures with fluorescent markers, such as a hybrid of gold nanoshells (NSs) and QDs.^48^ The localized surface plasmon resonance of NSs (with silica core and gold shell) can be tuned across the NIR region through adjustment of the dimensions of both the core and the shell. Depending on their size, such NSs can display either strong absorption or strong scattering, or both. We have shown that NSs with very thin gold shells exhibit strong 1PA and 2PA, thereby enabling applications in both bioimaging and photothermal treatment.^49^

Enhancement of 2PA cross section (σ_2_) in hybrid nanomaterials has been widely discussed in the literature, especially for combinations of plasmonic NPs with organic molecules^50−55^ and QDs.^56−58^ However, in most cases, the investigations have been conducted through two-photon excited photoluminescence. In such studies, it is challenging to determine the contribution of the radiative recombination rate enhancement (including the Purcell effect) compared to the actual increase of the σ_2_ values alone. Another method for estimating the σ_2_ value is the measurement of saturation of two-photon-excited photoluminescence. This technique involves recording the emission spectrum for different pump flux levels and then plotting the dependence of emission intensity on the square of the photon flux. The analysis of the obtained data allows for further calculations related to the σ_2_ value even for samples with low optical density. This method is highly sensitive; however, similar to the previous one, it is applicable only to samples that exhibit sufficiently bright two-photon-excited emission.^57,59,60^ In the research presented here, we measure the changes in transmittance as a function of light intensity using the Z-scan technique, thus assessing the enhancement of σ_2_ in a direct way. To demonstrate the possibility of developing low-toxicity hybrid nanomaterials with high σ_2_ values, we concentrate on colloidal systems containing hybrid nanostructures obtained by integrating Ag_2_S QDs with silica nanospheres, Au-NPs, and Au-NSs. Simultaneously, we perform frequency-domain electromagnetic simulations to assess the physical mechanisms responsible for the enhancement of NLO effects, such as local field enhancement and plasmon-exciton coupling (yielding a double peak in the σ_2_ spectrum). We compare the values of σ_2_/*M* for bare QDs and QDs integrated into the nanostructures, demonstrating vastly superior 2PA properties of the latter ones. In several cases the 2PA process competes with absorption saturation, which we also quantify. In one case we reveal the emergence of an additional NLO phenomenon, which should be formally treated as saturation of extinction driven by two-photon absorption process.

## 2 Materials and Methods

### 2.1 Synthesis

#### 2.1.1 Ag_2_S QDs

We synthesized 3-mercaptopropionic acid (3-MPA) modified Ag_2_S QDs using chemicals purchased from Merck. The synthesis was based on the procedure proposed by the Acar group,^61^ however, we implemented some modifications.^5^ Briefly, in two separate instances, solutions were prepared using 75 mL of deionized water for silver nitrate(V) with 3-MPA, and 25 mL of deionized water for sodium sulfide. In the next step, all the prepared reagents were deoxygenated, degassed under vacuum, and stored under nitrogen atmosphere. The reactions were carried out in a three-neck flask with nitrogen flow. Initially, 75 mL of water was added, followed by the introduction of 3-MPA, with the pH being set to 8.5. Afterward, silver nitrate(V) was added, and the pH was readjusted to 8.5 using acetic acid and sodium hydroxide. Heating was started in an oil bath equipped with a reflux condenser, while maintaining a nitrogen atmosphere at 90 °C. Once the target temperature was attained, a solution of sodium sulfide in 25 mL of water was carefully added drop by drop. The synthesis was allowed to proceed for 3 h with the reaction mixture continuously stirred. After the synthesis was completed and the product cooled, the resulting solution underwent filtration using a 0.22 μm syringe filter.

#### 2.1.2 Ag_2_S@SiO_2_

A bottle containing several mL of QDs solution was placed on a magnetic stirrer, and an excess of ethanol was introduced to alter the reaction environment. Subsequently, 1 mL of ammonia solution and 15 μL of TEOS (tetraethyl orthosilicate) were added. The resulting mixture was covered with aluminum foil and stirred overnight. Next, the solution underwent filtration through a 450 μm pore size filter to separate it from the precipitate. The filtrate underwent separation via high-speed centrifugation, and the supernatant was decanted. The precipitate was then dissolved in a small volume of water. The resulting aqueous solution was entirely utilized in the subsequent step. The solution of Ag_2_S@TEOS from the preceding step was completely transferred to a medium-sized flask placed on a magnetic stirrer. Subsequently, twice the volume of ethanol in comparison to water was added to alter the reaction environment. Finally, 150 μL of APTES ((3-aminopropyl)triethoxysilane) was introduced to the solution. The prepared mixture was covered with aluminum foil and left overnight with continuous stirring. Next, the flask underwent sonication until any precipitate dissolved, after which it was centrifuged with an additional portion of ethanol at low speed. The supernatant was separated from the precipitate, which was then dissolved in the same volume of ethanol added before centrifugation. The resulting solution is referred to as Ag_2_S@SiO_2_. Subsequently, solutions for modification with gold nanoparticles were prepared. THPC Gold: In a small beaker on a magnetic stirrer, several milliliters of water were mixed with 0.5 mL of THPC (tetrakis(hydroxymethyl)). Concurrently, in a large beaker on a stirrer, 200 mL of water and approximately 1 mL of 1 M NaOH were mixed. In the subsequent step, 33 mL of the THPC solution were added to the NaOH solution, and the mixture was stirred for several minutes. Following this, an equal volume of 1% gold chloride solution was rapidly added to the solution while stirring continued. Au-KCarb: 200 mL of water was introduced into a dark bottle, followed by the addition of 0.36 mol of K_2_CO_3_ and 3 mL of 1% gold chloride solution. The entire mixture was shaken and left in a dark place for aging.

#### 2.1.3 Ag_2_S@SiO_2__Au-islands

A bottle containing 20 mL of THPC Gold obtained in the previous step was placed on a magnetic stirrer. 0.5 mL of NaCl and 10 mL of Ag_2_S@SiO_2_ were added to the bottle. The mixture was left overnight, shielded from light with aluminum foil. The following day, the solution underwent sonication until any precipitate dissolved, and then it was centrifuged at low speeds. After decanting the supernatant, the precipitate was dissolved in water. Ag_2_S@SiO_2__Au: A plastic cuvette was filled to approximately 3/4 of its volume with Au-KCarb solution. Varied amounts of Ag_2_S@SiO_2__Au-islands, ranging from 10 to 100 μL, and a drop of formaldehyde were added. The mixture was shaken for several minutes. For the prepared solutions, the absorption spectrum was measured to select the optimal ratio of Au-KCarb solution to Ag_2_S@SiO_2__Au-islands. To obtain a larger quantity of Ag_2_S@SiO_2__Au-layer, proportionally larger amounts of reagents were added to a bottle placed on a magnetic stirrer and stirred intensively for a few minutes. The entire mixture underwent low-speed centrifugation, and the precipitate was dissolved in 10 mL of water.

#### 2.1.4 NSs

All chemicals were purchased from Sigma-Aldrich, except for the 120 nm diameter silica nanoparticles purchased from nanoComposix. The synthesis of NSs was performed following the method described by the Halas group^62−64^ with some adjustments introduced by our group.^49,65^ The procedure begins with the preparation of several solutions, which then need to be properly aged under different conditions. Initially, a 1% solution of tetrachloroauric(III) acid in deionized water was prepared and stored in darkness. Subsequently, tetrakis(hydroxymethyl)phosphonium chloride (80% in H_2_O), 1 M sodium hydroxide solution and aged 1% tetrachloroauric(III) acid solution were mixed to prepare THPC solution which was stored in a refrigerator until further use. Next, the plating solution was prepared: 50 mg of potassium carbonate was dissolved in 200 mL of deionized water, followed by the addition of 3 mL of aged gold chloride solution. In the following step, functionalization of the 120 nm silica nanospheres was conducted using APTES (99.99%): 7 mL of the silica nanospheres solution was diluted with deionized water, centrifuged twice at 3000 rcf for 25 min, suspended in ethanol, and injected with 400 μL of APTES, left stirring overnight, centrifuged twice, and suspended in ethanol. Subsequently, gold islands from the THPC solution were attached to APTES-functionalized silica spheres, referred to as seed solution. In the final stage, the plating solution, seed solution, and formaldehyde solution were rapidly mixed for 2 min. After adjusting the position of the extinction band, a solution of appropriate volume was prepared.

#### 2.1.5 NS@SiO_2_

The exact procedure has been described in our previous work, see ref (66). Briefly, in a separate glass container, 3 mL of the NSs colloidal solution was thoroughly mixed with 4 mL of deionized water. Ethanol, ammonia–water, and 4 μL of TEOS were added dropwise, and the mixture was left to slowly mix overnight. The suspension underwent two rounds of centrifugation and was then suspended in 10 mL of ethanol before being stored in the refrigerator.

#### 2.1.6 NS@SiO_2_ APTES

In the next stage, NSs with a silica layer (NS@SiO_2_) were functionalized using APTES. For this purpose, 25 μL of APTES was added to 10 mL of NS@SiO_2_ solution, left to mix overnight, and then centrifuged at 280 rcf for 20 min.

#### 2.1.7 NS@SiO_2_@Ag_2_S-QDs

Ag_2_S QDs were attached to the functionalized NSs. 200 μL of Ag_2_S QDs was added to 2 mL of NSs@SiO_2_ APTES solution, the container was wrapped in aluminum foil, and left to mix overnight. The mixture was centrifuged at 280 rcf for 20 min, and then dissolved in 5 mL of water or ethanol.

### 2.2 Experimental Methods

#### 2.2.1 TEM

For nanoparticle size characterization, a FEI Tecnai G^2^ 20 X-TWIN transmission electron microscope (TEM) was employed. The solutions were deposited onto TEM grids, dried, and then imaged.

#### 2.2.2 Spectroscopy

The absorption spectra of the solutions were measured in a 1 cm quartz cuvette using a Shimadzu UV-1900i spectrometer.

#### 2.2.3 Z-scan Technique

The NLO properties of the synthesized nanomaterials were investigated using the closed- and open-aperture Z-scan technique. A detailed description of the measurements can be found in our previous works.^28,67,68^ Briefly, a Coherent Astrella Ultrafast regenerative amplifier was used to generate laser pulses of appr. 55 fs duration, at 800 nm with 1 kHz repetition rate. The pulses served as the pump beam for a TOPAS-PRIME parametric amplifier which enabled λ tuning from 500 to 1600 nm. After suitable λ selection, the generated beam was focused to a focal spot with a waist *w*_0_ in the range 26–73 μm using a lens. The calculated intensity at the focal point varied from 100 to 390 GW/cm^2^. The pulse energies were approximately 1 μJ, corresponding to an average power of around 1 mW. Three silicon photodiodes (for the visible region) or InGaAs photodiodes (for the NIR region) were utilized for detection of the reference, open-aperture, and closed-aperture signals which were digitized using a National Instruments PCI-6143 I/O card, enabling simultaneous sampling across all the channels. The obtained data were subsequently transferred to a computer employing LabVIEW software. In accordance with prior investigations conducted by our group, the Z-scan data obtained for colloidal nanoparticle solutions at various λ were calibrated against closed-aperture measurements conducted on a 3 mm thick silica plate.^5,25,49,69^

### 2.3 Numerical Methods

#### 2.3.1 COMSOL Simulations

We performed numerical frequency-domain simulations of the optical scattering by the hybrid nanostructures using COMSOL Multiphysics, Wave Optics module. Three different types of nanostructures were considered: (1) silica core with 171 QDs uniformly distributed in the volume and 234 gold islands uniformly distributed on the surface, (2) the same silica core with QDs, but with a continuous gold shell instead of gold islands, (3) silica-coated gold nanoshell with a single QD on the surface. The results obtained for (1) and (2) are presented in the SI (Figures S6 and S7), while the results for (3) are shown in Figure 4 in the main article. In each case, the QDs were modeled as spherical nanoparticles of radius 1.38 nm and refractive index 2.2 (corresponding to that of bulk Ag_2_S), while the refractive indices of silica, gold, and water (the surrounding medium) were taken from the refractiveindex.info database.^70^ In case (3), we assumed the silica core radius of 60 nm, gold shell thicknesses 20 nm, and silica layer thickness 10 nm. In cases (1) and (2), the radius of the silica core was 63 nm, the radii of the gold islands (case (1)) were 1.5 nm, and the thickness of the gold shell (case (2)) was 8.5 nm. The maximum element size was set to 75 nm for the entire model and 7.5 nm for the gold shell in case (3). Otherwise, it was locally constrained by the small geometrical features (gold islands, QDs, thin gold shell). Each model geometry contained only one nanostructure, surrounded by a perfectly matched layer (PML), and the outer boundary condition was set as a scattering boundary condition with zero field. The models were solved for the scattered field, with background field defined as a linearly polarized plane wave. The Poynting vectors for both the scattered field and the total field were calculated on a spherical boundary enclosing the nanostructure (at a distance slightly closer than the PML), and the Poynting vector component normal to the boundary was integrated to yield the scattered and absorbed power, which were subsequently converted to normalized scattering and absorption cross sections (with extinction evaluated as the sum of the two).

It should be pointed out that the above method, in which the optical properties of gold nanoparticles and QDs are modeled using the refractive indices of bulk materials, is not fully accurate due to the breakdown of classical electromagnetic theory for nanoparticles of sizes below 10 nm.^71^ We also neglected the quantum confinement effects that are known to significantly modify the optical properties of QDs,^72^ as well as the effects of surface roughness^65^ and polydispersity in nanoparticles size.^66^ Despite these very coarse approximations, the obtained results provided useful insights into the electromagnetic mechanisms underlying the experimental observations, especially the spectral dependence of the NLO properties enhancement.

## 3 Results and Discussion

### 3.1 Nanomaterials Studied in This Work

**Figure 1:**
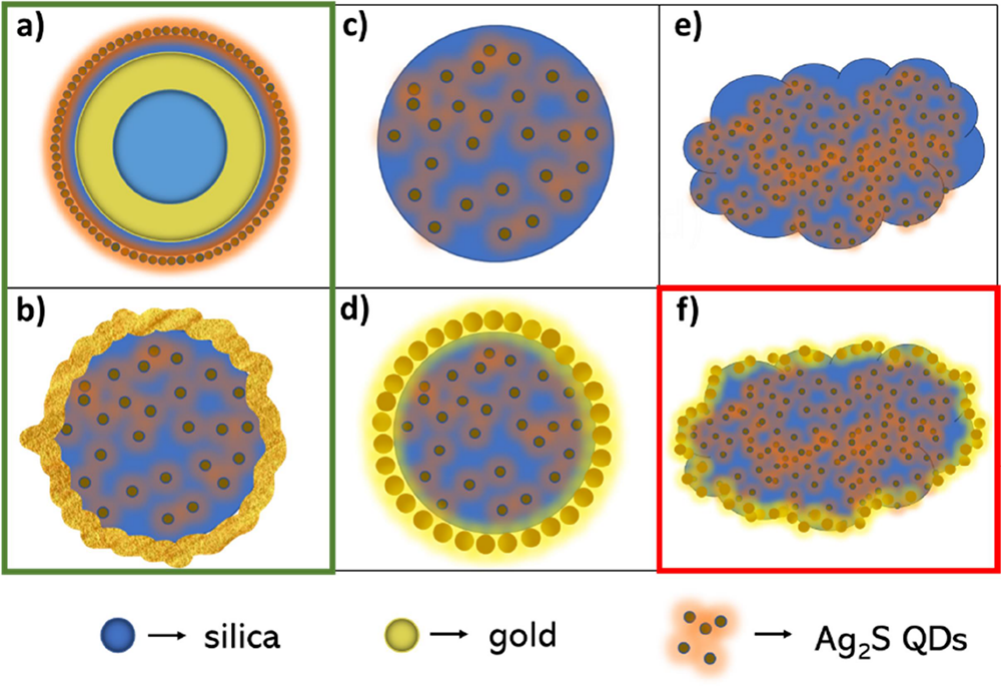
*S*chematic illustration of the six types of hybrid nanomaterials studied in this work: (a) Au nanoshells with silica spacer layer, decorated with Ag_2_S QDs (NS@SiO_2_@Ag_2_S-QDs); (b) Ag_2_S QDs with 2-MPA ligand, embedded in silica nanospheres covered by a continuous gold layer (Ag_2_S_2MPA@SiO_2__Au-layer); (c, e) Ag_2_S QDs embedded in silica nanospheres (Ag_2_S@SiO_2_). Two different ligands, 2-MPA and 3-MPA, were used for the QDs; thus, the nanostructures are also referred to as Ag_2_S_2MPA@SiO_2_ and Ag_2_S_3MPA@SiO_2_; (d, f) Ag_2_S QDs embedded in silica (Ag_2_S_2MPA@SiO_2_ or Ag_2_S_3MPA@SiO_2_), covered by gold islands (Ag_2_S_2MPA@SiO_2__Au-islands or Ag_2_S_3MPA@SiO_2__Au-islands). Note: Structures on the left (inside green frame) are discussed in the main text, while the other structures are presented in the SI. The NLO properties of all the structures were investigated using Z-scan, except the one inside the red frame, which was not measured due to its rapid sedimentation.

In this work, we have obtained six types of hybrid nanomaterials, which are shown schematically in Figure 1. In the main text, we mainly focus on the structures shown in Figure 1a,b (marked by the green contour), which are Au nanoshells decorated with Ag_2_S QDs (NS@SiO_2_@Ag_2_S-QDs) and Au nanoshells with Ag_2_S QDs embedded in the core (Ag_2_S_2MPA@SiO_2__Au-layer). These structures were found to exhibit the most interesting NLO properties. The other structures are only briefly introduced in the main text as the products of intermediate synthesis steps (Figure 1c,d) or the structures resulting from using an alternative surface ligand (Figure 1e,f). More details about their properties can be found in the SI.

### 3.2 General Aspects of Comparing NLO Properties of Different Nanomaterials

Several caveats need to be discussed before presenting the actual data obtained in the measurements. To quantitatively assess the enhancements of the NLO properties of hybrid nanostructures that may be considered a synergistic effect, the results should be compared to the separate contributions of the components. Silica itself does not bring any meaningful contribution to the nonlinear absorption effects, but its presence regulates the interaction between the QDs and the plasmonic components of the nanostructures. For Ag_2_S NPs, we have previously observed a moderately strong 2PA, as well as saturable absorption at shorter λ. Ag_2_S QDs functionalized with 3-MPA exhibited 1PA saturation in the 500–770 nm range, and 2PA in the 800–930 nm range.^5^ On the other hand, it is well-known that NLO effects in metal nanostructures are strongly dependent on their shapes and sizes. The particular case of Au-NSs has been investigated in ref. (49) The nonlinear absorption in the NSs is mostly dominated by absorption saturation, although a contribution of 2PA could be seen at relatively short λ. We demonstrated that the NSs with a thin gold shell (10 and 13 nm) exhibit 2PA in the spectral range from 530 to 570 nm.^49^ The highest 2PA cross section was observed for NSs with a 10 nm gold shell. Increasing the shell thickness led to a decrease of 2PA accompanied by an increasing contribution of 1PA saturation. In the present study, we utilized NSs with the gold thickness of 20 nm, for which previously we have not observed any 2PA, only 1PA saturation from 600 to 900 nm.^49^

The quantification of the nonlinear absorption enhancement in a hybrid nanostructure needs to take into account different sizes of the investigated objects, for which, as discussed in refs (73, 74) a helpful quantity is σ_2_/*M*, which is relevant when considering applications of 2PA. One should note, however, that the enhancement can also be referred to the contribution of the QDs alone, by considering how much bigger is σ_2_ of a QD being a part of a hybrid structure compared to the σ_2_ of a single isolated QD. This can be assessed by dividing σ_2_ of a nanostructure by the total weight of the QDs incorporated in it and comparing that to σ_2_/*M* of an isolated QD. In other words, the relevant factor for discussing the enhancement will then be the quantity σ_2_/(*Mx*_QD_), where *x*_QD_ is the weight fraction of the QDs in the hybrid structure. In the present work *x*_QD_ was typically around 0.03, which means that the values of σ_2_/*M* enhancement should be multiplied by roughly a factor of 30.

Accurate evaluation of both σ_2_/*M* and σ_2_/(*Mx*_QD_) requires rigorous and precise determination of *M*. In this work, *M* was estimated based on the nanoparticle size obtained from TEM images as well as taking into account the proportions and densities of the chemical components of the nanomaterials. Determination of *M* was done at each stage of the synthesis to minimize errors in estimating *M* for the final hybrid nanomaterials.

Moreover, we should emphasize that for all the studied samples, the measurements are carried out in the spectral ranges where 1PA (which can come from both the QDs and the plasmonic structures) is not negligible. Thus, one cannot rule out the possibility of some contribution of absorption from the excited state (sequential absorption of two photons) to the 2PA results. Hence, we refer to the measured cross section as an effective 2PA cross section (σ_2_^eff^).

On the other hand, the phenomenon of absorption saturation is best evaluated in terms of the saturation intensity *I*_sat_, i.e., the intensity at which the absorption coefficient is reduced to half of its unsaturated value. This parameter should in principle be independent of concentration but the interpretation of its variation upon modifications of the absorbing nano-objects is not straightforward. Simple models (see the SI) predict that the reciprocal of *I*_sat_ should be proportional to the absorption cross section, thus, we usually plot *I*_sat_^–1^ as a function of λ and compare this spectrum to that of absorbance.

### 3.3 Ag_2_S QDs Embedded In Silica Nanospheres (Ag_2_S_2MPA@SiO_2_ and Ag_2_S_3MPA@SiO_2_) and Decorated by Gold Islands (Ag_2_S_2MPA@SiO_2__Au-islands and Ag_2_S_3MPA@SiO_2__Au-islands)

In this section, we summarize the NLO properties of Ag_2_S_2MPA@SiO_2_, Ag_2_S_3MPA@SiO_2_, and Ag_2_S_2MPA@SiO_2__Au-islands. These structures were also used as intermediates to synthesize other products, namely, Ag_2_S_2MPA@SiO_2__Au-layer and NS@SiO_2_@Ag_2_S-QDs (which are discussed further in the text).

Detailed characterization of the samples Ag_2_S_2MPA@SiO_2_, Ag_2_S_3MPA@SiO_2_, Ag_2_S_2MPA@SiO_2__Au-islands, and Ag_2_S_3MPA@SiO_2__Au-islands is presented in the SI. Figures S1 and S2 provide the TEM images, while Figure S5 shows the measured extinction spectra. Numerically calculated extinction, scattering, and absorption spectra are presented in Figure S6, whereas Figure S7 shows the modeled geometries along with numerical simulations of the local field intensity distributions.

The spectral dependence of σ_2_ for Ag_2_S_2MPA@SiO_2_, Ag_2_S_3MPA@SiO_2_, and Ag_2_S_2MPA@SiO_2__Au-islands is depicted in Figure S3. A comprehensive summary of these results is available in Tables 1 and S1, with detailed explanations and supplementary data provided in the SI. Briefly, the analysis reveals that the σ_2_/*M* for Ag_2_S_2MPA@SiO_2_ reaches a value of 0.087 GM·mol·g^–1^ at 900 nm, approximately seven times higher than that of bare QDs. In contrast, Ag_2_S_3MPA@SiO_2_ exhibits the σ_2_/*M* value of 0.039 GM·mol·g^–1^ at 925 nm. This reduction in performance, compared to Ag_2_S_2MPA@SiO_2_, can be attributed to morphological differences between the two nanostructures. Notably, when adjusting for the QD weight fraction, i.e. considering σ_2_/(*Mx*_QD_), the enhancement for Ag_2_S_2MPA@SiO_2_ approaches nearly 2 orders of magnitude, underscoring the potential for weight-specific optimization. A similar enhancement effect has been observed in silica-embedded organic dyes,^75,76^ suggesting broader applicability in optimizing nanosystems for two-photon absorption using silica. The NLO properties are further augmented in Ag_2_S_2MPA@SiO_2__Au-islands, which demonstrates absorption saturation in the visible range (see Figure S3c) and enhanced 2PA in the near-infrared, with σ_2_/*M* reaching 0.592 GM·mol·g^–1^ at 875 nm, an order of magnitude higher than that of Ag_2_S_2MPA@SiO_2_. This significant improvement is attributable to the local electric field enhancement induced by the gold island plasmon resonance, which is further supported by numerical simulations (see Figure S7b) and corroborated by prior studies.^65^

**Table 1:** Average and Maximum Enhancements of σ_2_/*M* and σ_2_/(*Mx*_QD_) for Hybrid Materials^a^

sample name	average enhancement of σ_2_/*M*	maximum enhancement of σ_2_/*M*	average enhancement of σ_2_/(*Mx*_QD_)	maximum enhancement of σ_2_/(*Mx*_QD_)
Ag_2_S_2MPA@SiO_2_	7	16 at 900 nm	157	412 at 900 nm
Ag_2_S_3MPA@SiO_2_	3	6 at 900 nm	82	142 at 900 nm
Ag_2_S_2MPA@SiO_2__Au-islands	42	73 at 900 nm	829	1447 at 900 nm
NS@SiO_2_@Ag_2_S-QDs	12	67 at 750 nm	375	2137 at 750 nm

a Ag_2_S_2MPA@SiO_2_, Ag_2_S_3MPA@SiO_2_, Ag_2_S_2MPA@SiO_2__Au-islands, and NS@SiO_2_@Ag_2_S-QDs compared to the peak values for the bare Ag_2_S QDs.

### 3.4 NLO Properties of Ag_2_S_2MPA@SiO_2__Au-layer

**Figure 2:**
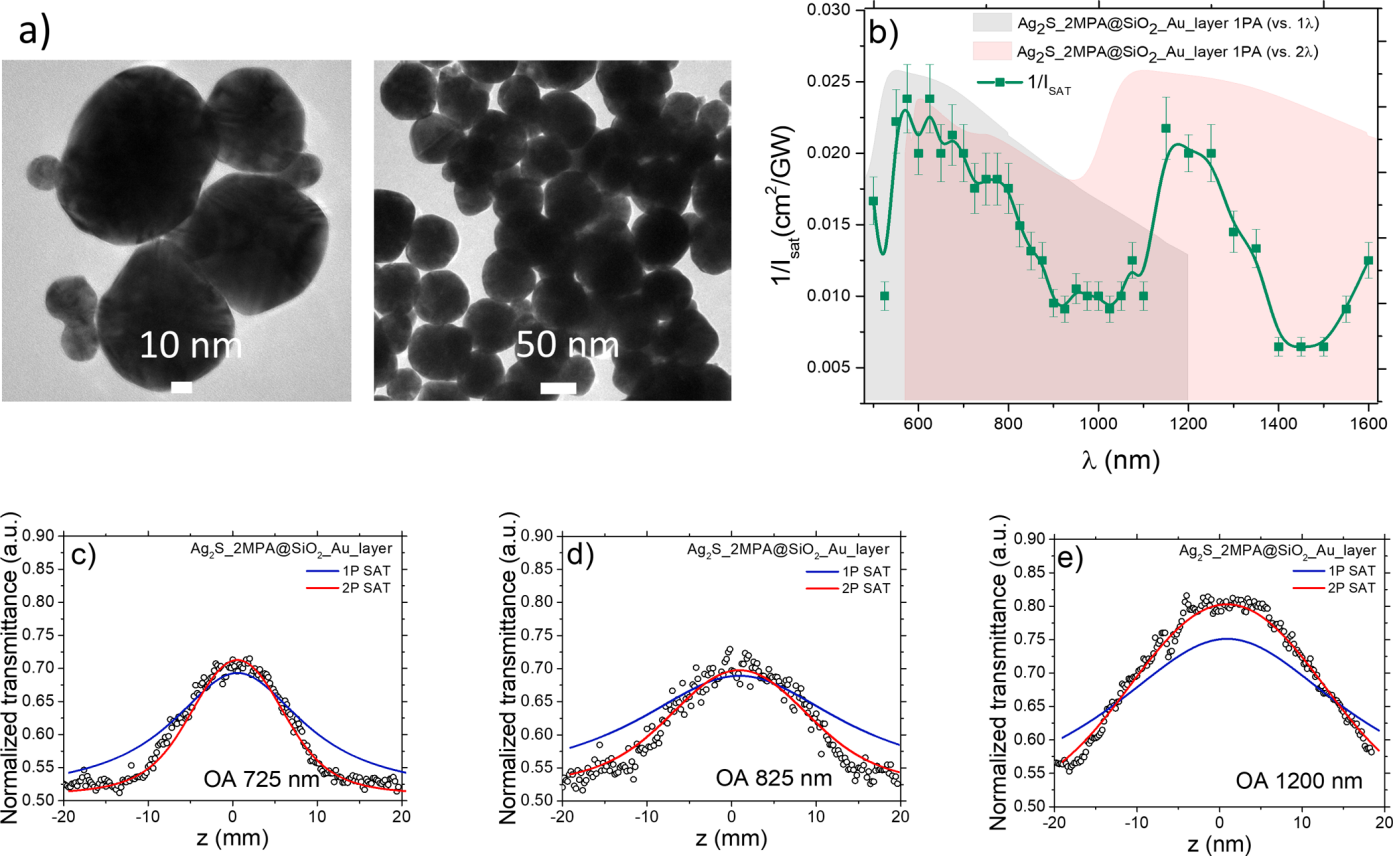
(a) TEM images of the nanostructures labeled as Ag_2_S_2MPA@SiO_2__Au-layer. (b) Spectral dependence of 1/*I*_sat_ corresponding to extinction saturation (green squares, the green solid line is used to guide the eye) for Ag_2_S_2MPA@SiO_2__Au-layer. The gray-shaded area shows the measured extinction spectrum plotted vs 1λ, while the pink-shaded area corresponds to the same spectrum plotted vs 2λ. The coincidence of the two bands observed in the 1/*I*_sat_ spectrum with the two versions of the extinction spectrum may indicate the involvement of both one-photon (1P) and two-photon (2P) driving of the saturation effects. Representative open-aperture Z-scan traces obtained for Ag_2_S_2MPA@SiO_2__Au-layer at (c) 725 nm, (d) 825 nm, and (e) 1200 nm. The blue curves correspond to the conventional 1PA saturation model (1P SAT), while the red curves correspond to the proposed 2PA-driven saturation (2P SAT). The excellent fit obtained with the latter model suggests that 2PA-driven extinction saturation is the likely mechanism underlying the observed transmittance variation.

The Ag_2_S_2MPA@SiO_2__Au-layer sample was obtained by coating the surface of Ag_2_S_2MPA@SiO_2__Au-islands with a uniform gold layer of an average thickness of 8.5 nm (see the TEM images in Figure 2a, the measured extinction spectra in Figure S5a, and the model geometry in Figure S7c). In the case of Ag_2_S_2MPA@SiO_2__Au-layer, the Z-scan measurements showed very distinct saturation effects across the entire investigated spectral range (see Figure 2b). However, the attempts to fit the open-aperture Z-scan curves using the conventional nonlinear absorption theory (see eqs SI1 and SI2) were not successful (see Figure 2c–e, blue lines). Upon searching for alternate expressions, we found that excellent fits could be obtained if the extinction coefficient was assumed to obey the following functional dependence (a simple derivation of this saturation law is given in the SI):
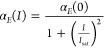
1where α_*E*_(*I*) stands for the intensity dependent extinction coefficient. We stress here the replacement of the term “saturable absorption” with “saturable extinction” because, in fact, the 1PA cross sections are smaller than the scattering cross sections within most of the spectral range where the saturation is observed. We used eq 1) to fit the experimental curves (see Figure 2c–e, red lines), which has resulted in the spectral dependence of 1/*I*_sat_ showing a broad band following the extinction spectrum of the investigated system plotted vs λ and another band corresponding roughly to the same spectrum replotted vs 2λ (see Figure 2b). This may serve as an indication that the saturation mechanism does involve absorption of two photons, at least in the range 1000–1400 nm. However, clearly, this is different from 2PA saturation considered in the literature, which was studied, e.g., in transition metal dichalcogenides.^77^ In our case, there is no direct indication of a 2PA process, which normally would give a dip in the open-aperture Z-scan curve. Instead, the extinction is dominated by scattering and the increase of transmitted intensity clearly must be attributed either to weakening of the scattering or the increase of transmittance by some alternative means, e.g., stimulated emission. We note that the derivation of the saturation law presented in the SI predicts the proportionality of  values to those of σ_2_, however, at present there is no sufficient information on the kinetics of the processes involved to explore this relation in a quantitative way. Time-resolved studies would be helpful to shed more light on the mechanism of this unusual saturation in the NIR region.

### 3.5 Gold Nanoshells with Silica Spacer Layer, Decorated by Ag_2_S QDs (NS@SiO_2_@Ag_2_S-QDs)

In creating another type of hybrid nanostructures with enhanced NLO properties, we used gold nanoshells (NSs) with the extinction band tuned to the NIR range and having balanced contributions of absorption and scattering. In the first series of experiments, we employed NSs with silica cores of 60 nm radius and gold shells of 20 nm thickness. Having in mind potential future use of the studied nanostructures as two-photon excited fluorescent nanolabels, we decided to separate the QDs from the gold surface of the NSs by a thin dielectric spacer. Such spacers between the emitter and the metal are used to limit quenching of the excited fluorophores, which otherwise would occur in consequence of nonradiative energy transfer to the metal surface. Commonly used spacer materials are, for example, proteins,^78^ PEG-SH,^65^ or silica.^66^ Among these options, we have chosen silica due to its relatively easy thickness control, durability, and the potential for further surface modifications. We obtained a silica layer of around 10 nm thickness. In the final stage, the Ag_2_S QDs were attached to the surface, forming a monolayer of QDs, each QD having an average diameter of 2.76 nm. The synthesis procedure is described in detail in section “Materials and methods”, whereas schematic illustration is provided in Figure S8.

**Figure 3:**
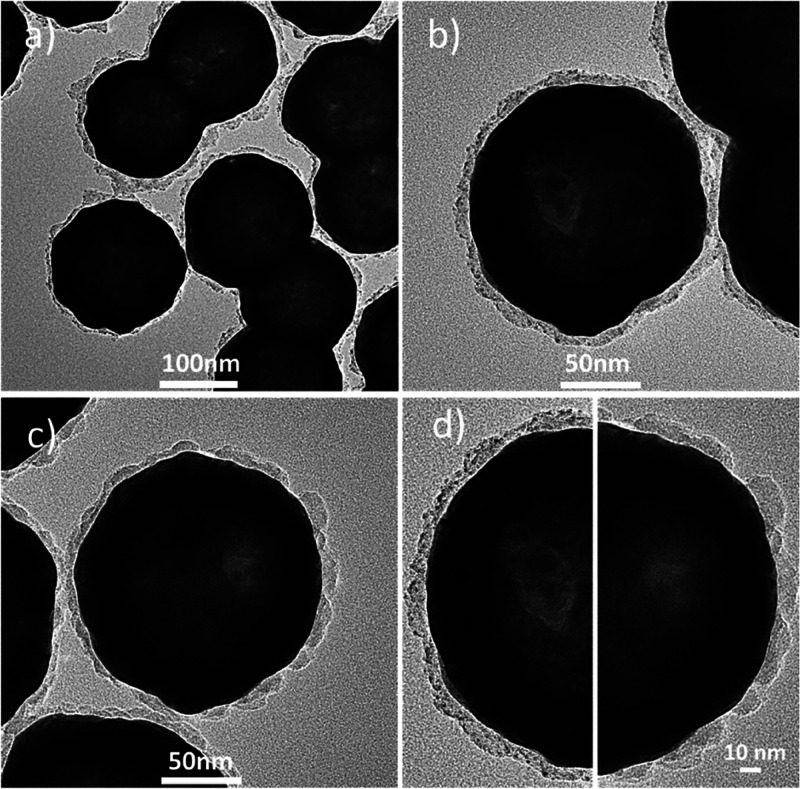
TEM images of the NSs covered with a silica layer. (a, b) Silica layer is functionalized with Ag_2_S QDs, which are visible as darker dots on the silica surface (NS@SiO_2_@Ag_2_S-QDs). For comparison, the QDs are absent in (c) (NS@SiO_2_). (d) Two halves of a NS are compared. Left: NS coated with a silica layer and Ag_2_S QDs (NS@SiO_2_@Ag_2_S-QDs). Right: NS with the silica layer only (NS@SiO_2_).

The TEM images of the obtained nanostructures are shown in Figure 3a,b, revealing nearly monodisperse NSs with the silica layer and QDs on the surface. For comparison, Figure 3c shows a TEM image of the NSs with the silica layer only. A direct comparison of NSs with and without QDs is shown at a higher magnification in Figure 3d.

Figure S9 compares the extinction spectra of a colloidal solution of NSs (black curve) and the same NSs with the additional layer of silica and Ag_2_S QDs (blue curve), labeled as NS@SiO_2_@Ag_2_S-QDs. Upon attachment of silica and QDs, a redshift of the extinction band by approximately 11 nm is observed. This behavior results from the sensitivity of the localized surface plasmon resonance to the refractive index of the environment, and it has also been observed in our previous works.^65,66^

**Figure 4:**
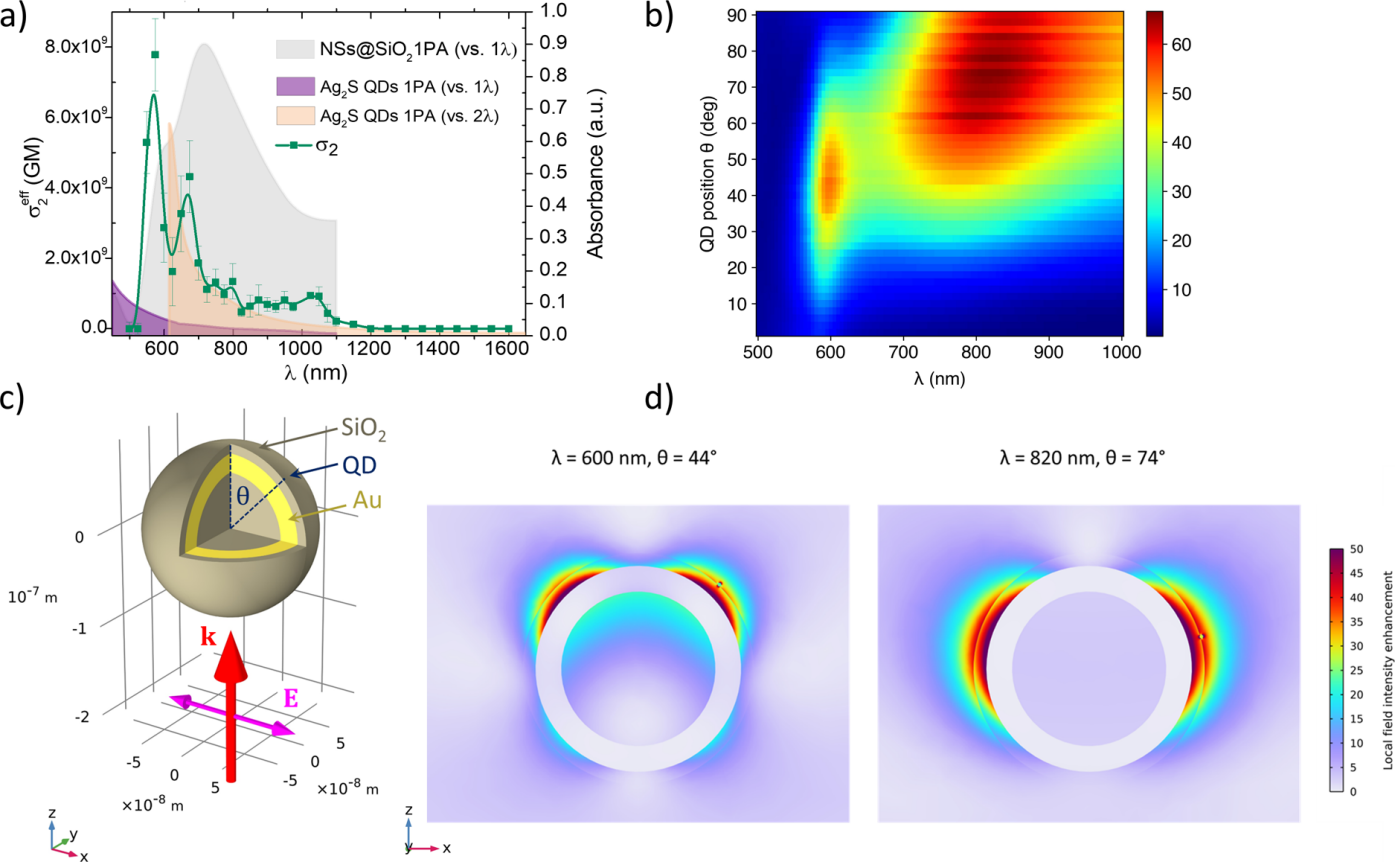
(a) Spectral dependence of 2PA cross section (σ_2_^eff^) measured using the Z-scan technique for the colloidal solution NS@SiO_2_@Ag_2_S-QDs. The values of σ_2_^eff^ are plotted as green filled squares (the green solid line is used to guide the eye). The gray area represents the measured 1PA spectrum of NSs, the purple area represents the measured 1PA spectrum of Ag_2_S QDs vs 1λ, and the beige area represents the same 1PA spectrum plotted vs 2λ. (b) Numerical simulations of the local electric field intensity enhancement around the QD as a function of λ and angle θ. (c) Schematic illustration of the numerically modeled NS and the illumination geometry, with the red arrow indicating the propagation direction of the incident light, and the magenta double-arrow showing the incident light polarization. The model includes only one QD attached to the NS surface. The QD radius is 1.38 nm, and its refractive index is 2.2. The location of the QD with respect to the incident light is defined by the polar angle θ. (d) Representative plots of the local field intensity distribution at the quadrupole (left) and dipole resonance (right). In both cases, the plots are obtained for the QD located in one of the plasmonic hot spots.

Figure 4a shows the spectral dependence of σ_2_ for a colloidal solution of NS@SiO_2_@Ag_2_S-QDs. 2PA could be detected over a broad λ range from 550 to 1150 nm, the spectrum exhibiting two maxima, the first at λ of 575 nm with σ_2_ = 7.78 × 10^9^ GM and the second at 675 nm with σ_2_ = 4.32 × 10^9^ GM. No saturable absorption was observed in this sample.

The normalized cross section σ_2_/*M* for NS@SiO_2_@Ag_2_S-QDs can be calculated from the data in Figure 4a, estimating *M* to be 1.79 × 10^10^ g·mol^–1^ for a hybrid NP (in comparison, an individual Ag_2_S QD has *M* = 4.79 × 10^4^ g·mol^–1^). Consequently, σ_2_/*M* at 575 nm is 0.436 (GM·mol·g^–1^), whereas at 675 nm, it is 0.242 (GM·mol·g^–1^) and decreases down to 0.046 at 875 nm. All these values are much higher than the peak value for the bare Ag_2_S QDs with 2-MPA, which was only 0.013 (GM·mol·g^–1^) at 850 nm,^5^ even though the QDs are only a small fraction (around 0.03) of the total weight of a hybrid NP.

The observed significant enhancement of the normalized 2PA cross section is attributable to the plasmonic local field enhancement and, possibly, also to coupling between the localized surface plasmon resonance and the QD’s exciton.^57,79,80^ One could hypothesize that such coupling could lead to Rabi splitting, being responsible for the double peak in the σ_2_ spectrum. Large values of σ_2_ coincide with the spectral regions of significant local electric field enhancement revealed by numerical simulations (see Figure 4b,d). In these simulations, the field enhancement is investigated depending on the QD position on the NS surface with respect to the light incidence direction (quantified by polar angle θ). Figure 4b shows the enhancement due to the electric dipole resonance (with maximum around 820 nm) and electric quadrupole resonance (with maximum around 600 nm). The dipole resonance leads to the electric field enhancement mainly on the sides of the NS (θ close to 90°, see example field distribution in Figure 4d on the right). On the other hand, the quadrupole resonance gives rise to the enhancement in the areas between the sides and the back of the NS (θ around 45°, see example field distribution in Figure 4d on the left). Since the QDs are uniformly distributed on the NSs surface, the effect of the local field enhancement in the form of increased 2PA cross sections is visible at both resonances. However, if the 2PA enhancement involves coupling between plasmon and QD exciton, then one should expect the quadrupole mode, having a higher quality factor than the dipole mode, to play a dominant role. Large enhancement of σ_2_ and double-peak line shape in Figure 4a in the spectral range corresponding to that of the quadrupole resonance suggest that such coupling and the associated Rabi splitting may indeed be the underlying mechanism in this case. However, our theoretical model does not account for the surface roughness and nonuniformity in nanoparticles size, both of which may have a significant effect on the experimentally observed optical properties.^65,66^ On the experimental side, the double-peak in Figure 4a is barely resolved by our Z-scan measurements. Still, the presented σ_2_ values were obtained from a rigorous Z-scan analysis with well-defined error bars, which means that the observed double-peak is not an artifact. Combined with the spectral coincidence of this peak with the quadrupole plasmon resonance, the evidence for plasmon-exciton coupling in the σ_2_ spectrum is rather strong. Our results show that hybrid nanomaterials combining Ag_2_S QDs and Au NSs exhibit intriguing NLO properties that deserve further theoretical and experimental investigation.

## 4 Summary and Conclusions

In this work, we have combined Ag_2_S QDs with silica and gold nanostructures, creating a series of hybrid nanomaterials with strongly enhanced NLO properties. The schematic summary of the results is presented in Figure S10 and the measured values are given in Tables 1 and S1. In the case of NSs with QDs embedded in the silica core, we have observed an unusual process of extinction saturation. By fitting the Z-scan trace shapes with a modified theoretical model, we have discovered that the observed extinction saturation may involve two-photon absorption as the driving process. We have also discovered that attaching Ag_2_S QDs to the surface of NSs, as well as embedding these QDs into silica nanospheres decorated with gold islands, can lead to strong enhancements of the 2PA process, manifested as a significant increase in σ_2_/*M* compared to the bare QDs. These enhancements can be considered even higher if one treats them simplistically as the influence of the surroundings on the cross section of a single QD, i.e., takes into account the σ_2_/(*Mx*_QD_) merit factor. Indeed, enhancements of σ_2_/(*Mx*_QD_) by more than 3 orders of magnitude are seen, see Tables 1 and S1. However, these values should be taken with some reserve since, in fact, both QDs and metal structures can contribute to the overall nonlinear absorption. Finally, numerical simulations have shown that the enhanced nonlinear effects and their spectral variation can be attributed to the interaction of QDs with the local optical fields enhanced by the surface plasmon resonances and, possibly, also to coupling between plasmon modes and QD excitons, which may be responsible for the observed double peak in the σ_2_ spectrum. Our results demonstrate that hybrid nanomaterials combining quantum-confined, dielectric and plasmonic components can exhibit vastly superior NLO properties, thus having a large potential for two-photon excited bioimaging, as well as theranostic and optical signal processing applications. Simultaneously, the interplay between various components of the nanostructures can give rise to unusual NLO effects that are fundamentally interesting and deserve further investigation. Last but not least, of particular interest are the emission properties of the Ag_2_S QDs-Au NS hybrid nanomaterials, which are a target of our ongoing research.

## Supporting Information Available

Morphology description and TEM images of Ag_2_S_2MPA@SiO_2_, Ag_2_S_3MPA@SiO_2_, Ag_2_S_2MPA@SiO_2__Au-islands, Ag_2_S_3MPA@SiO_2__Au-islands, and Ag_2_S_3MPA@SiO_2__Au-layer; discussion of the spectral dependence of σ_2_^eff^ and 1/*I*_sat_ (reciprocal of 1PA saturation intensity) for Ag_2_S_2MPA@SiO_2_, Ag_2_S_3MPA@SiO_2_, and Ag_2_S_2MPA@SiO_2__Au-islands; measured and numerically calculated extinction spectra for Ag_2_S_2MPA@SiO_2__Au-islands and Ag_2_S_2MPA@SiO_2__Au-layer, supplemented by the numerical simulations of the local field intensity distribution; illustration of the process of creating NS@SiO_2_@Ag_2_S_QD; measured extinction spectra for the colloidal solutions of NSs and NS@SiO_2_@Ag_2_S-QDs; summary of the NLO effects observed in the studied nanostructures; derivation of the quadratic intensity dependence of the extinction saturation; and supplementary table presenting the experimentally obtained values of two-photon absorption cross section σ_2_ and the related merit factors σ_2_/*M* and σ_2_/(*Mx*_QD_) (PDF)
