# Supplementary material for: Strong Enhancement of Two-Photon Absorption and Emergence of Unusual Extinction Saturation in Silver Sulfide Quantum Dots Integrated with Gold and Silica Nanostructures
Source: ACS Appl Mater Interfaces. 2025 May 3;17(19):28484–94. doi: 10.1021/acsami.5c00984 (PMC12086773; doi:10.1021/acsami.5c00984)
Supplement: Supplementary file 1 — am5c00984_si_001.pdf [file am5c00984_si_001.pdf]

Supporting Information

Strong Enhancement of Two-photon Absorption and Emergence of Unusual Extinction Saturation in Silver Sulfide Quantum Dots Integrated with Gold and Silica Nanostructures

Marta Gordel-Wójcik,[a*] Radosław Kołkowski,[b] Marcin Nyk,[c] Marek Samoć[c*]

[a]Faculty of Chemistry, University of Wrocław, 14.p F. Joliot-Curie Street, 50-383, Wrocław, Poland

[b]Department of Applied Physics, Aalto University, P.O.Box 13500, FI-00076 Aalto, Finland

[c]Institute of Advanced Materials, Faculty of Chemistry, Wrocław University of Science and Technology, Wyb. Wyspiańskiego 27, PL-50370, Wrocław, Poland

corresponding authors:

Marta Gordel-Wójcik (marta.gordel-wojcik@uwr.edu.pl),

Marek Samoć (marek.samoc@pwr.edu.pl).

The following Supporting Information file contains:

1. Morphology description and TEM images of *Ag_2S_2MPA@SiO_2* and *Ag_2S_3MPA@SiO_2* (Supplementary Figure S1).
2. Morphology description and TEM images of *Ag_2S_2MPA@SiO_2_Au-islands* and *Ag_2S_3MPA@SiO_2_Au-islands* (Supplementary Figure S2).
3. Discussion of the spectral dependence of σ_2^{eff} and 1/Isat (reciprocal of 1PA saturation intensity) for *Ag_2S_2MPA@SiO_2*, *Ag_2S_3MPA@SiO_2*, and *Ag_2S_2MPA@SiO_2_Au-islands* (Supplementary Figure S3).
4. Morphology description and TEM images of Ag2S_3MPA@SiO2_Au-layer (Supplementary Figure S4).

5. Measured (Supplementary Figure S5) and numerically calculated extinction spectra (Supplementary Figure S6) for *Ag_2S_2MPA@SiO_2_Au-islands* and *Ag_2S_2MPA@SiO_2_Au-layer*, supplemented by the numerical simulations of the local field intensity distribution (Supplementary Figure S7).
6. Illustration of the process of creating *NS@SiO_2@Ag_2S_QD* (Supplementary Figure S8).
7. Measured extinction spectra for the colloidal solutions of NSs and *NS@SiO_2@Ag_2S-QDs* (Supplementary Figure S9)
8. Summary of the nonlinear optical effects observed in the studied nanostructures (Supplementary Figure S10).
9. Derivation of the quadratic intensity dependence of the extinction saturation.
10. Supplementary Table S1 presenting the experimentally obtained values of two-photon absorption cross section σ_2 and the related merit factors σ_2/M and σ_2/(Mx_{QD}).

1. Ag_2S QDs embedded in silica nanospheres (*Ag_2S_2MPA@SiO_2* and *Ag_2S_3MPA@SiO_2*)

These were nanostructures in which the Ag_2S QDs reside within the interiors of silica nanoparticles rather than on their surfaces, which were intermediaries for obtaining more complex nanostructures containing gold. First, we synthesized the QDs in a silica-based medium (see Fig. S1 a). We found that the resulting nanomaterial was different depending on the functionalization of Ag_2S QDs. For the QDs functionalized with 2-mercaptopropionic acid (2-MPA), we obtained nanostructures possessing a uniform spherical shape of diameter 63 nm ± 7.3 nm (see Figs. S1 b and S1 c, named as *Ag_2S_2MPA@SiO_2*). In contrast, functionalization with 3-mercaptopropionic acid (3-MPA) yielded non-uniform nanomaterial with dimensions on the micrometer scale (see Figs. S1 d and S1 e, named as *Ag_2S_3MPA@SiO_2*).[2]

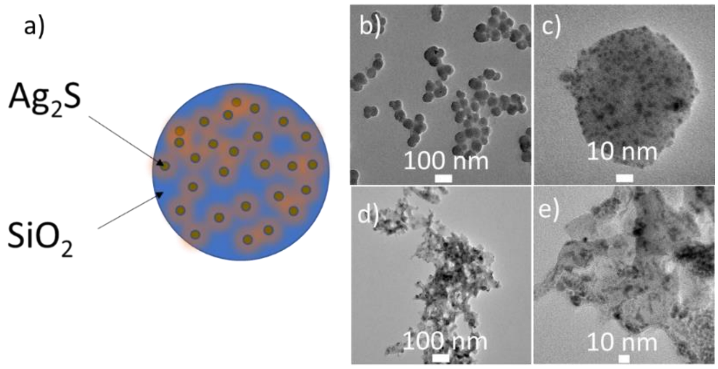

Fig. S1 *a) Illustration of a nanoparticle composed of Ag_2S QDs embedded in silica ($Ag_2S@SiO_2$); b),c) TEM images of $Ag_2S@SiO_2$ based on QDs functionalized with 2-MPA (Ag_2S_2MPA@SiO_2); d),e) TEM images of $Ag_2S@SiO_2$ based on QDs functionalized with 3-MPA (Ag_2S_3MPA@SiO_2).*

2. Ag_2S QDs embedded in silica nanospheres (*$Ag_2S@SiO_2$*), decorated by gold islands (*Ag_2S_2MPA@SiO_2_Au-islands* and *Ag_2S_3MPA@SiO_2_Au-islands*)

In the next step, we attached gold islands to the previously obtained silica-QDs nanostructures, naming them as *Ag_2S_2MPA@SiO_2_Au-islands* for nanostructures based on Ag_2S QDs with 2-MPA, and *Ag_2S_3MPA@SiO_2_Au-islands* for nanostructures based on Ag_2S QDs with 3-MPA (see TEM images in Fig. S2).

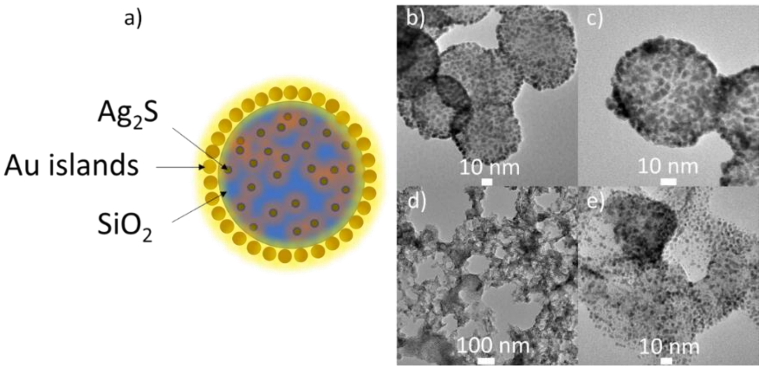

Fig. S2 *a) Illustration of $Ag_2S@SiO_2$_Au-islands, obtained by decorating $Ag_2S@SiO_2$ with gold islands; b), c) TEM images of $Ag_2S@SiO_2$_Au-islands based on QDs functionalized with 2-MPA (Ag_2S_2MPA@SiO_2_Au-islands); d), e) TEM images of $Ag_2S@SiO_2$_Au-islands based on QDs functionalized with 3-MPA (Ag_2S_3MPA@SiO_2_Au-islands).*

3. Comparison of the NLO spectra of *Ag_2S_2MPA@SiO_2*, *Ag_2S_3MPA@SiO_2* and *Ag_2S_2MPA@SiO_2_Au-islands*

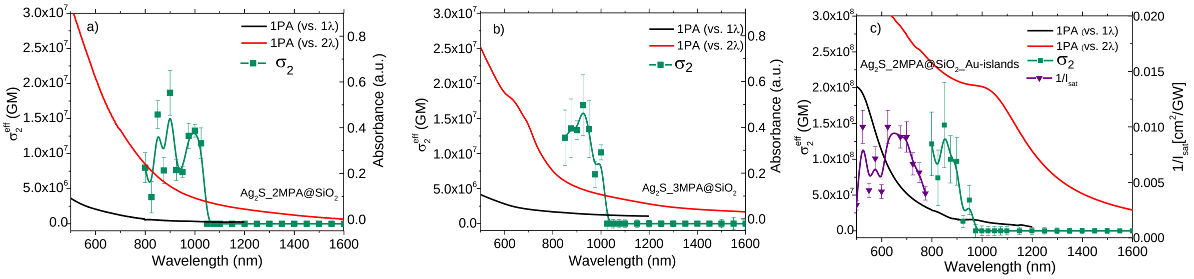

***Fig. S3** Spectral dependence of σ_2^{eff} and $1/I_{sat}$ (reciprocal of 1PA saturation intensity) for Ag_2S_2MPA@SiO_2 (a) (see TEM images in Fig S1 b, c), Ag_2S_3MPA@SiO_2 (b) (see TEM images in Fig S1 d, e) and Ag_2S_2MPA@SiO_2_Au-islands (c) (see TEM pictures in Fig. S2 b, c). The σ_2^{eff} values are plotted as green filled squares (the green solid line is used to guide the eye). The values of $1/I_{sat}$ are plotted as purple filled triangles (the purple line is used to guide the eye). The black line represents the 1PA spectrum plotted vs. λ, and the red line represents the same 1PA spectrum plotted vs. 2λ.*

Similarly to the bare Ag_2S QDs studied in our earlier work, both *Ag_2S_2MPA@SiO_2* and *Ag_2S_3MPA@SiO_2* show 2PA around 800 nm, which is experimentally observed as a transmittance dip in the open-aperture Z-scan curves measured in this spectral range. The 2PA range is slightly broader in *Ag_2S_2MPA@SiO_2* (750 - 950 nm) compared to that in *Ag_2S_3MPA@SiO_2* (800 - 900 nm).[1] While the magnitudes of σ_2 of colloidal Ag_2S QDs functionalized with 2-MPA and 3-MPA showed little difference[1], there are greater differences for the QDs embedded in silica. This can be analysed in terms of σ_2/M, which reaches 0.087 (GM·mol·g^{-1}) at 900 nm for *Ag_2S_2MPA@SiO_2*. This value is around 7 times greater than that of bare QDs, see Tables 1 and S1. A smaller value, σ_2/M = 0.039 (GM·mol·g^{-1}) was measured at 925 nm for *Ag_2S_3MPA@SiO_2*, which may be attributed to the morphological differences of the two materials. On the other hand, employing the $\sigma_2/(Mx_{QD})$ factor, which accounts for the fact that the QDs constitute only a few percent of the total weight of the hybrid species, one concludes that the actual enhancement defined this way is as large as approximately two orders of magnitude. We note that the enhancement of 2PA and two-photon excited emission have also been reported for organic dyes embedded into silica nanoparticles[2,3] and more detailed investigations of this effect may lead to nanosystems optimized for efficient 2PA.

The results of the Z-scan measurements of *Ag_2S_2MPA@SiO_2_Au-islands* are presented in Fig. S3 c. Notably, the colloidal solution of these nanostructures shows absorption saturation effects in the visible range and 2PA in the near-infrared range. Similarly to the approach used in our earlier studies on silica spheres covered with gold islands,[4] we describe the process of light absorption in such samples by the following equation:

$$\frac{dI}{dz} = -\alpha_{SA} I - \alpha_2 I^2 \qquad \text{(SI 1)}$$

where α_{SA} (saturable 1PA coefficient) is defined by the following expression, which is usually applied for the case of saturation of homogeneously broadened absorption:

$$\alpha_{SA} = \frac{\alpha_0}{1+\frac{I}{I_{sat}}} \qquad \text{(SI 2)}$$

In addition to the emergence of 1PA saturation in *Ag_2S_2MPA@SiO_2_Au-islands*, the sample shows σ_2/M reaching 0.592 (GM·mol·g^{-1}) at 875 nm, which is an order of magnitude higher than that for *Ag_2S_2MPA@SiO_2*. Consequently, also the $\sigma_2/(Mx_{QD})$ factor is significantly increased, see Tab. S1. This improvement in the 2PA merit factors can be attributed to the electric field enhancement on the surface of the nanostructure by gold islands, which is investigated by numerical simulations presented further in this SI document. The underlying mechanism may involve local electric field enhancement by the plasmon resonance of the gold islands. This is consistent with our previous studies, which have shown that an incomplete layer of gold on a silica sphere strongly enhances the electric field of the incident light.[5]

4. Ag_2S QDs embedded in silica (*Ag_2S@SiO_2*), covered by gold layer (*Ag_2S_3MPA@SiO_2_Au-layer*)

Further modification of the previously obtained nanomaterials involved coating them with a continuous gold layer. *Ag_2S_2MPA@SiO_2_Au-islands* were successfully coated with a layer of gold of average thickness 8.5 nm, yielding a new type of nanostructures labelled as *Ag_2S_2MPA@SiO_2_Au-layer* (see Fig. 2 a). However, in the case of *Ag_2S_3MPA@SiO_2_Au-islands*, a complete gold layer was not formed; instead, there was only an increase in the diameter of the gold islands (see TEM images in Fig. S4). Nevertheless, we labelled the new nanomaterial as *Ag_2S_3MPA@SiO_2_Au-layer* for consistency. Due to the rapid sedimentation of its colloidal solution, this material could not be studied using Z-scan.

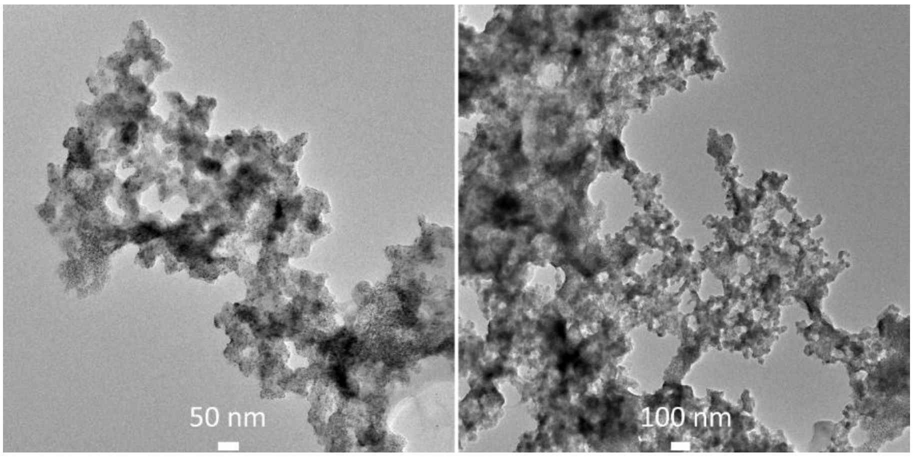

Fig. S4 *TEM images at various magnifications, showing the micrometer-sized structures labelled as Ag_2S_3MPA@SiO_2_Au-layer, obtained after additional gold deposition on the surface of Ag_2S_3MPA@SiO_2_Au-islands. In this case, the formation of a continuous gold layer on the surface was not successful, and only separated gold nanoparticles of increased size are observed in the new nanomaterial.*

5. Measured extinction spectra and numerical simulations for *Ag_2S_2MPA@SiO_2_Au-islands* and *Ag_2S_2MPA@SiO_2_Au-layer*.

The extinction spectra measured for *Ag_2S_2MPA@SiO_2, Ag_2S_2MPA@SiO_2_Au-islands* and *Ag_2S_2MPA@SiO_2_Au-layer* are presented in Fig. S5. To identify the features observed in these spectra, we have numerically simulated the optical response of the corresponding nanostructures. The modelled geometries are idealized and neglect the inherent polydispersity of the experimental samples, which is why the calculated line shapes are far from perfect agreement with the measured ones. Nevertheless, a rough correspondence between the measured and calculated plasmon resonance peaks has been established.

Figure S6 a shows numerically calculated absorption, scattering, and extinction cross sections, normalized by the geometrical cross sections, of a silica core of radius 63 nm, containing 171 Ag_2S QDs in the volume and 234 gold islands on the surface (corresponding to the nanostructures *Ag_2S_2MPA@SiO_2_Au_ islands* previously presented in the TEM images in Fig. S2 b and c; the model geometry is shown in Fig. S7 a). The calculated absorption spectrum shows only one sharp peak at 540 nm, at which the absorption cross section is significantly larger than the scattering cross section. The local field intensity distribution at this wavelength

is presented in Fig. S7 b. The scattering spectrum essentially follows the Rayleigh scattering theory ($1/\lambda^4$ dependence), except for the small bulge due to the aforementioned resonance, which can also be observed in the measured extinction spectrum (red curve in Fig. S5 a).

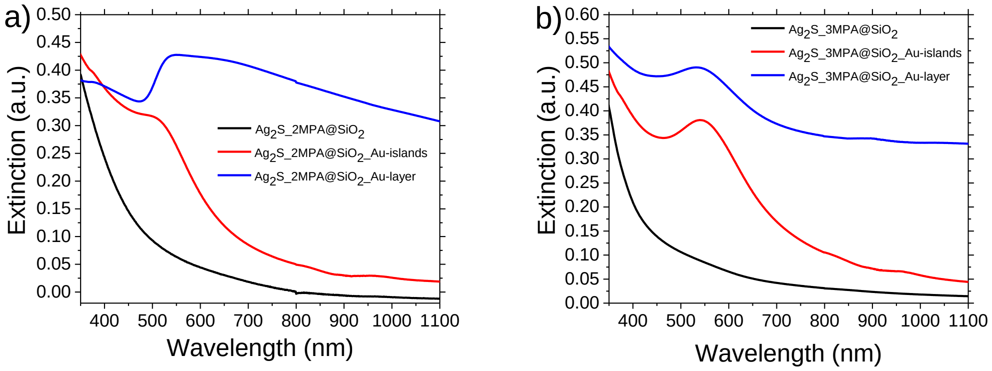

Fig. S5 *Measured extinction spectra for the nanostructures based on Ag_2S QDs functionalized with 2-MPA (a) and 3-MPA (b).*

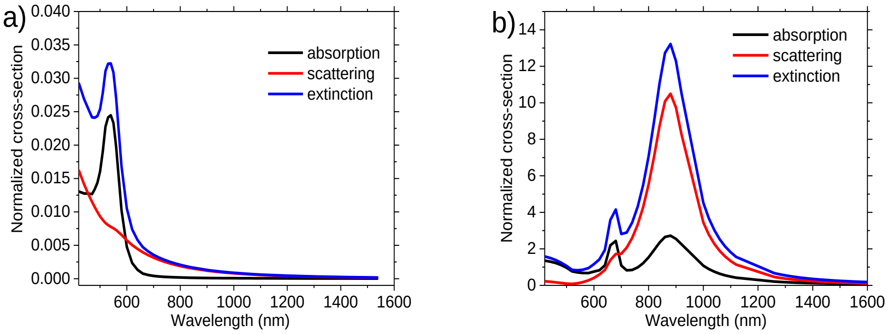

Fig. S6 *Numerically calculated spectra of the extinction (blue line), scattering (red line) and absorption (black like) for a) Ag_2S_2MPA@SiO_2_Au_ islands, b) Ag_2S_2MPA@SiO_2_Au-layer.*

Figure S6 b shows the calculated normalized absorption, scattering, and extinction cross sections of a nanoparticle with the same QD-doped silica core as in the previous case, but covered with a continuous gold shell of thickness 8.5 nm instead of gold islands (corresponding to *Ag_2S_2MPA@SiO_2_Au-layer*, see TEM images in Fig. 2 a; the model geometry is shown in Fig. S7 c). The absorption spectrum shows a very sharp peak at 670 nm, corresponding to the electric quadrupole resonance of the gold nanoshell. Due to its subradiant nature, the quadrupole resonance is associated with strong enhancement of absorption cross section compared to scattering cross section at the resonance wavelength. On the other hand, the second peak at 880 nm corresponds to the electric dipole resonance associated with large

scattering cross section, which makes it significantly broader due to radiative damping. Inhomogeneous broadening of both resonances is most likely responsible for the broad extinction band observed in the measured extinction spectrum (blue curve in Fig. S5 a). Local field intensity distribution at the quadrupole (left) and dipole resonance (right) for this nanostructure is presented in Fig. S7 d.

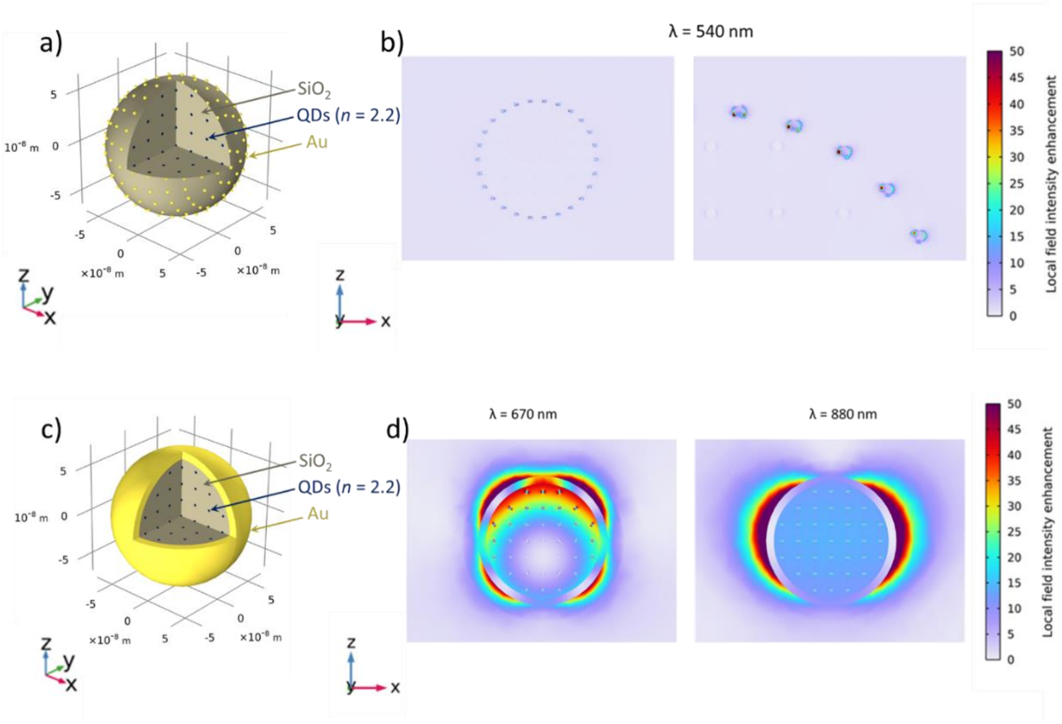

Fig. S7 *Numerical simulations corresponding to Ag_2S_2MPA@SiO_2_Au-islands and Ag_2S_2MPA@SiO_2_Au-layer. a) Model geometry – silica core with QDs embedded in the volume and gold islands attached to the surface. The silica core radius is 63 nm. The core contains 171 QDs that are uniformly distributed in the volume of the core. Each quantum dot is modelled as a sphere of radius 1.38 nm and refractive index 2.2. There are also 234 gold islands uniformly distributed on the silica surface. Each gold island has a radius of 1.5 nm. Periodic arrangement of the QDs in the core does not have any effect on the optical properties of the nanostructures due to their deeply subwavelength dimensions, which prevents the appearance of diffraction artifacts. b) Local field intensity distribution at λ = 540 nm, corresponding to the resonance visible in the spectrum in Fig. S6 a. c) Model geometry with QDs embedded in the silica core (as in a)) but with a continuous shell of thickness 8.5 nm instead of gold islands. d) Local field intensity distribution at the quadrupole (left) and dipole resonance (right), which are visible in the spectra in Fig. S6 b.*

6. Illustration of the process of creating *NS@SiO$_2$@Ag$_2$S-QD.*

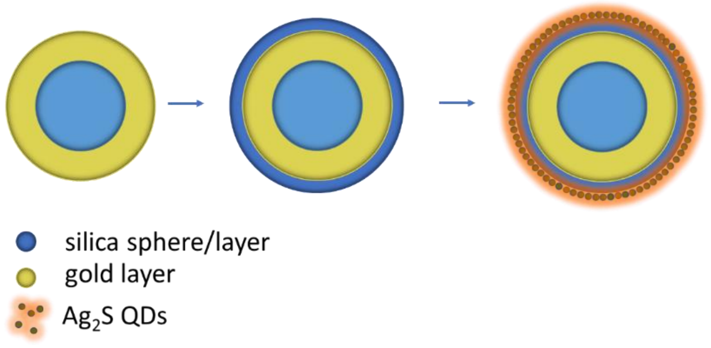

Fig. S8 *Illustration of the process of creating a hybrid material based on the NSs and Ag$_2$S QDs. First, the NSs are formed, consisting of a silica sphere and a gold layer. In the subsequent step, a silica layer serving as a spacer is created. In the final stage, Ag$_2$S QDs are attached to the silica layer.*

7. Measured extinction spectra for NSs and *NS@SiO$_2$@Ag$_2$S-QDs*.

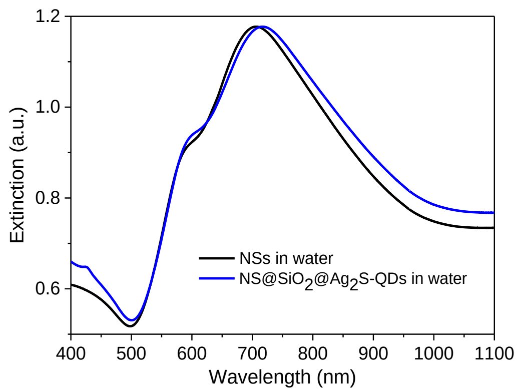

Fig. S9 *Extinction spectra of the colloidal solutions in water, containing NSs (black line) and NSs with the silica layer (10 nm) decorated with Ag$_2$S QDs (NS@SiO$_2$@Ag$_2$S-QDs, blue line).*

8. Summary of the NLO properties of all the nanomaterials studied in this work.

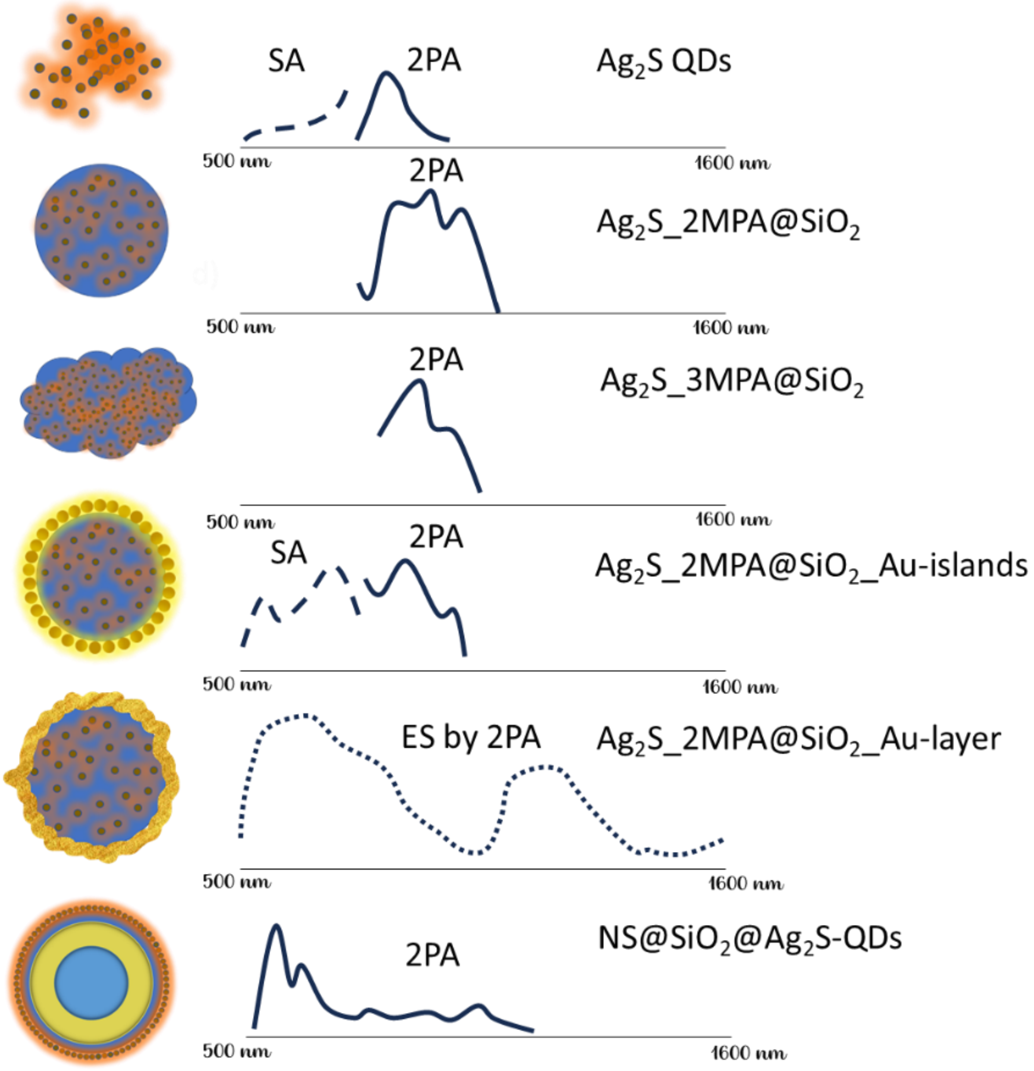

Fig. S10 *Illustration of the nonlinear optical processes observed in the studied nanomaterials. 2PA stands for two-photon absorption, SA for saturable absorption, and ES by 2PA indicates saturable extinction driven by two-photon absorption.*

9. Quadratic intensity-dependent extinction saturation

We follow here the simple kinetic rate equation model that in the case of one-photon absorption leads to Eq. (SI 1). If two-photon absorption is present (simultaneous absorption of two photons or a sequential process) and dominates over the 1PA, then the population of an excited state is given by

$$\frac{dN_1}{dt} = (N_0 - N_1)\sigma_2\Phi^2 - \frac{N_1}{\tau} ,$$

where N_0 is the population of the ground state, N_1 is the population of an excited state, σ_2 is the two-photon absorption cross section, Φ is the photon flux and τ is the excited state lifetime. Assuming steady-state approximation leads to:

$$N_1 = (N_0 - N_1)\sigma_2\tau\Phi^2 \, ,$$

$$N_1(1 + \sigma_2\tau\Phi^2) = N_0\sigma_2\tau\Phi^2,$$

$$N_1 = N_0\sigma_2 \frac{\tau\Phi^2}{(1 + \sigma_2\tau\Phi^2)} \, .$$

The one-photon extinction coefficient is assumed to be given by

$$\alpha_E(I) = (N_0 - N_1)(\sigma_1 + \sigma_S) = (N_0 - N_1)\sigma_E$$

where σ_1, σ_S and σ_E are the absorption, scattering and extinction cross sections, respectively. Therefore:

$$\alpha_E(I) = N_0 \left[1 - \sigma_2 \frac{\tau\Phi^2}{(1+\sigma_2\tau\Phi^2)}\right] \sigma_E = N_0 \left[\frac{1}{(1+\sigma_2\tau\Phi^2)}\right] \sigma_E \, .$$

Since

$$\Phi = \frac{I}{h\nu} \, ,$$

we arrive at

$$\alpha_E(I) = \frac{\alpha_E(0)}{1 + \left(\frac{I}{I_{sat}}\right)^2} \, ,$$

where

$$I_{sat} = \frac{(h\nu)^2}{\sigma_2\tau} \, .$$

10. Experimentally obtained values of σ_2, σ_2/M and $\sigma_2/(Mx_{QD})$.

Table S1. *Summary of the nonlinear optical properties of hybrid nanomaterials exhibiting 2PA: Ag_2S_2MPA@SiO_2, Ag_2S_3MPA@SiO_2, Ag_2S_2MPA@SiO_2_Au-islands, and NS@SiO_2@Ag_2S-QDs. Data in the table:* ***σ_2*** *in 10^6 GM;* ***σ_2/M*** *in GM·mol·g^{-1};* ***average and maximum enhancement of σ_2/M****;* ***$\sigma_2/(Mx_{QD})$*** *in GM·mol·g^{-1} (this value is σ_2 divided by the fraction of Ag_2S QDs in the total molar mass of the hybrid structure);* ***average and maximum enhancement of $\sigma_2/(Mx_{QD})$****.*

Ag_2S_2MPA@SiO_2					
Wavelength	σ_2	σ_2/M	Average and **maximum** enhancement of σ_2/M	$\sigma_2/(Mx_{QD})$	Average and **maximum** enhancement of $\sigma_2/(Mx_{QD})$
nm	10^6 GM	GM·mol·g^{-1}		GM·mol·g^{-1}	
800	7.99	0.037		0.96	
825	3.80	0.018		0.46	
850	15.57	0.073		1.88	
875	7.61	0.036		0.92	
900	18.66	0.087	7.1	2.25	157
925	7.65	0.036	**15.9**	0.92	**412**
950	7.37	0.034		0.89	
975	12.53	0.059		1.51	
1000	13.27	0.062		1.60	
1025	11.47	0.054		1.38	

Ag_2S_3MPA@SiO_2					
Wavelength	σ_2	σ_2/M	Average and **maximum** enhancement of σ_2/M	$\sigma_2/(Mx_{QD})$	Average and **maximum** enhancement of $\sigma_2/(Mx_{QD})$
nm	10^6 GM	GM·mol·g^{-1}		GM·mol·g^{-1}	
850	12.25	0.029		0.74	
875	13.59	0.032		0.82	
900	13.34	0.031		0.80	
925	16.90	0.039	3.2<br>**5.5**	1.02	82<br>**142**
950	13.48	0.031		0.81	
975	7.03	0.016		0.42	
1000	10.16	0.024		0.61	

Ag_2S_2MPA@SiO_2_Au-islands					
Wavelength	σ_2	σ_2/M	Average and **maximum** enhancement of σ_2/M	$\sigma_2/(Mx_{QD})$	Average and **maximum** enhancement of $\sigma_2/(Mx_{QD})$
nm	10^6 GM	GM·mol·g^{-1}		GM·mol·g^{-1}	
825	121.49	0.486		9.6	
850	74.06	0.296		5.9	
875	147.71	0.591		11.7	
900	99.92	0.400	41.9<br>**73.1**	7.9	829<br>**1447**
925	97.06	0.388		7.7	
950	13.33	0.053		1.1	
975	42.96	0.172		3.4	

NS@SiO₂@Ag₂S-QDs					
Wavelength	σ_2	σ_2/M	Average and **maximum** enhancement of σ_2/M	$\sigma_2/(Mx_{QD})$	Average and **maximum** enhancement of $\sigma_2/(Mx_{QD})$
nm	10^6 GM	GM·mol·g^{-1}		GM·mol·g^{-1}	
550	5288.17	0.296	11.7<br>**66.5**	9.5	375<br>**2137**
575	7784.16	0.436		14.0	
600	2870.79	0.161		5.2	
625	1620.07	0.091		2.9	
650	3265.12	0.183		5.9	
675	4321.05	0.242		7.8	
700	1864.33	0.104		3.4	
725	1116.72	0.062		2.0	
750	1317.34	0.074		2.4	
775	970.86	0.054		1.7	
800	1343.52	0.075		2.4	
825	477.45	0.027		0.9	
850	641.62	0.036		1.2	
875	813.72	0.046		1.5	
900	689.49	0.039		1.2	
925	631.24	0.035		1.1	
950	814.61	0.046		1.5	
975	624.10	0.035		1.1	
1025	946.27	0.053		1.7	
1050	919.80	0.051		1.7	
1075	437.42	0.024		0.8	
1100	210.00	0.012		0.4	
1150	122.05	0.007		0.2	

References:

(1) Gordel-Wójcik, M.; Malik, M.; Siomra, A.; Samoć, M.; Nyk, M. Third-Order Nonlinear Optical Properties of Aqueous Silver Sulfide Quantum Dots. *Journal of Physical Chemistry Letters* **2023**, *14* (49), 11117–11124. https://doi.org/10.1021/acs.jpclett.3c02820.

(2) Wu, W.-B.; Liu, C.; Wang, M.-L.; Huang, W.; Zhou, S.-R.; Jiang, W.; Sun, Y.-M.; Cui, Y.-P.; Xu, C.-X. Uniform Silica Nanoparticles Encapsulating Two-Photon Absorbing Fluorescent Dye. *Journal of Solid State Chemistry* **2009**, *182* (4), 862 – 868. https://doi.org/10.1016/j.jssc.2008.12.003.

(3) Aparicio-Ixta, L.; Ramos-Ortiz, G.; Pichardo-Molina, J. L.; Maldonado, J. L.; Rodríguez, M.; Tellez-Lopez, V. M.; Martinez-Fong, D.; Zolotukhin, M. G.; Fomine, S.; Meneses-Nava, Marco. A.; Barbosa-García, O. Two-Photon Excited Fluorescence of Silica Nanoparticles Loaded with a Fluorene-Based Monomer and Its Cross-Conjugated Polymer: Their Application to Cell Imaging. *Nanoscale* **2012**, *4* (24), 7751–7759. https://doi.org/10.1039/C2NR31925J.

(4) Gordel, M.; Olesiak-Banska, J.; Kolkowski, R.; Matczyszyn, K.; Buckle, M.; Samoc, M. Shell-Thickness-Dependent Nonlinear Optical Properties of Colloidal Gold Nanoshells. *Journal of Materials Chemistry C* **2014**, *2* (35), 7239–7246. https://doi.org/10.1039/c4tc01210k.

(5) Gordel-Wójcik, M.; Piela, K.; Kołkowski, R. Monitoring the Gold Nanoshell Growth Mechanism: Stabilizing and Destabilizing Effects of PEG-SH Molecules. *Physical Chemistry Chemical Physics* **2022**, *24* (9), 5700–5709. https://doi.org/10.1039/d2cp00239f.
